# Suicide ideation and behavior disparities among high school students: Examining Asian and multiracial race/ethnicity groups

**DOI:** 10.1371/journal.pmen.0000052

**Published:** 2024-07-02

**Authors:** Sugy Choi, Ji Eun Chang

**Affiliations:** 1 Department of Population Health, New York University Grossman School of Medicine, New York, New York, United States of America; 2 Department of Public Health Policy and Management, New York University School of Public Health, New York, NY, United States of America; Federal University of Sergipe: Universidade Federal de Sergipe, BRAZIL

## Abstract

We examined racial and ethnic disparities of suicide ideation/behaviors of high school students using detailed race/ethnicity data including Asian and multiracial race/ethnicity groups. The 2017 and 2019 Youth Risk Behavior Surveys were used to assess suicide ideation/behavior disparities across traditionally studied non-Latinx Black, Latinx, and non-Latinx White and understudied (non-Latinx Asian, multiracial-Latinx, multiracial-non-Latinx) racial/ethnic groups while controlling for common risk factors. Non-Latinx Black, multiracial Latinx, and multiracial non-Latinx students have higher odds of suicide attempt and injurious suicide attempt compared to non-Latinx Whites. Asian and multiracial non-Latinx students have higher odds of reporting suicide ideation and making a suicide plan compared to non-Latinx Whites. Results suggest that suicide ideation and behavior patterns differed considerably across racial/ethnic groups, and elicited the importance of including other racial/ethnic groups when conducting racial disparities analysis.

## Introduction

Suicide is the second-leading cause of death among high-school-aged youth in the United States, accounting for a third of injury-related deaths among this age group [1]. Suicide ideation/behavior may be an important indicator of mental distress that may be predictive of subsequent suicide [2]. Racial and ethnic groups face differential cultural and/or social risks for both suicide ideation (thinking about ending one’s life) and suicide attempts (non-fatal suicidal behavior). Yet, relatively little is known about the relationship between suicidal ideation/behaviors and race/ethnicity.

The fastest-growing racial/ethnic group in the U.S. is the multi-racial population, followed by Asian Americans [3]. Yet, research on suicide ideation and behavior among these populations have been unreported or kept on the margins [3–5]. A systematic review [5] examining ethnic differences in suicidal ideation and attempts found that most studies examining this topic collapse non-Black or Latinx racial minority groups into a single category [6], precluding the examination of suicidal ideation and behavior in those groups independently. Such limitations in data collection and reporting may limit our understanding of racial/ethnic differences in suicidal ideation and behaviors, especially for understudied groups.

Furthermore, while a growing body of literature have examined the broader health and behavior risk problems of mixed-race adolescents, these studies have often been based on clinical samples that have been self-selected for problems, precluding generalizability, or examined results of mixed-race samples without comparison to single-race groups, making it harder to assess differential risk between groups [7–10]. One exception is a 2018 study that compared the health and risk status of adolescents who identify with one race with those identifying with mixed race [11]. This study found that mixed race adolescents are at higher health and behavioral risk when compared to single-race counterparts. However, this study only included a single binary measure of suicide ideation and fell short of examining the differential risk and protective factors associated with the full range of suicide ideation and behaviors [12].

Nuanced examinations of racial/ethnic disparities in suicidal ideation and behaviors among more commonly studied populations such as White, Black, and Latinx youth have been inconclusive, with some studies suggesting higher suicide ideation/behavior risks among non-Latinx whites and other studies finding no significant relationship [1, 4, 13, 14]. A trend analysis reveals that Black students exhibited positive linear trends for suicide attempts regardless of sex, with Black adolescent boys also showing a significant linear increase in injury by attempt [15]. However, these studies have not examined the social determinants within which these students are embedded in depth. Additionally, these mixed results may be driven by the omission of key risk factors that are strongly associated with suicide attempts across different populations, including sexual minority status [13, 16], prior experience of bullying victimization [17], depression [18], having a good night sleep [19], and gender [20].

Given these gaps, the aim of our study was to identify and analyze the prevalence and correlates of suicidal ideation and behaviors across different racial/ethnic groups among high school students, using more nuanced racial/ethnic categorizations and controlling for a variety of demographic and psychosocial variables to enhance the understanding of these complex interactions. We examined racial/ethnic disparities in suicide ideation and suicide behavior including suicide plan making, suicide attempts, and injurious suicide attempts among high school students in 2017 and 2019 from the biannual Youth Risk Behavior Survey (YRBS). We used additional groupings of race/ethnicity data that have not been reported in the past and controlled for other underlying risk factors such as gender, sexual identity, or being bullied. Understanding racial/ethnic differences in suicidal ideation/behaviors is essential for more effectively directing suicide prevention efforts and tailoring interventions in culturally appropriate ways.

## Methods

This study uses two waves of the YRBS, a nationally representative, cross-sectional, and biannual survey of students in the United States [21]. The YRBS monitors six categories of health-related behaviors that contribute to the leading causes of death and disability among youth and adults including suicide ideation/behavior. The 2017 and 2019 waves included 28,442 respondents, with a response rate of 60.0 and 60.3 percent respectively. Analysis incorporated complex survey design variables and weights calculated by CDC to account for nonresponse and oversampling of Black and Latinx students [22]. There were 1,214 observations (∼4%) of the sample excluded due to missing grade level covariates or very small numbers within race/ethnicity subgroups (American Indian/Alaska Native (1%) and Native Hawaiian/Other Pacific Island groups (<1%).

### Ethical statement

In line with the policies of the NYU University Institutional Review Board (IRB), we did not obtain IRB approval because we used publicly available, de-identified data. While this article does not involve direct studies with human participants or animals by the authors, any procedures related to human participants adhere to ethical standards set by institutional or national research committees, as well as the 1964 Helsinki Declaration and its subsequent amendments or equivalent ethical standards.

### Measures

#### Race/ethnicity

Participants were classified into 6 groups: non-Latinx White, non-Latinx Black, Latinx, non-Latinx Asian, multiracial-Latinx, multiracial-non-Latinx.

#### Suicide ideation/behavior

We included four binary measures of suicidal ideation and behavior: considered suicide; made a suicide plan; attempted suicide; and injurious suicide attempts during the past 12 months. Survey questions can be found elsewhere [21].

#### Covariates

Covariates include gender (male/female), sexual orientation (heterosexual/Lesbian, Gay, bisexual/questioning), grade level (9^th^, 10^th^, 11^th^, or 12^th^), survey year, receipt of good grades (A’s/B’s) in school (yes/no), bullied on school property (yes/no), cyberbullied (yes/no), feeling sad or hopeless (yes/no), having at least 8 hours of sleep (yes/no).

#### Statistical analysis

We performed two multivariable logistic regressions using the STATA’s *svy* command. Model 1 examined the association between race/ethnicity and suicide ideation/behavior variables. Model 2 examined these associations after adding other risk factors previously known to be associated with suicide ideation/behavior, such as, receipt of good grades, experienced bullying on school property, cyberbullying, feeling sad or hopeless, and having a good night sleep. Listwise deletion handled missing data.

## Results

The analytical sample was composed of majority non-Latinx White (53%). Racial minority group representation varied (non-Latinx Black, 13%; Latinx, 10%; non-Latinx Asian, 4%; multiracial-Latinx, 15%; and multiracial-non-Latinx, 5%). Descriptive statistics are presented in S1 Table. Table 1 presents weighted multivariable analyses results. The results for 1 show that the multiracial-non-Latinx group estimates are at the highest odds of all suicide ideation/behavior outcomes. The findings are not significantly different from all non-White and Latinx students. Adding other risk factors in Model 2 elicited effects for non-Latinx Asians, who report higher suicide ideation and plan-making compared to non-Latinx White students. Students feeling sad and hopeless had the highest odds of suicide ideation/behavior outcomes. Receiving good grades in school is protective across all suicide ideation/behavior outcomes and experiencing bullying puts students at higher odds of negative suicide ideation/behavior outcomes. Accounting for additional factors attenuated the relationships between gender/sexual identity and suicide ideation/behavior.

**Table 1:** Race/ethnicity, gender, sexual identity, and risk factors associated with suicide ideation and behavior*.

Variables	Suicide ideation AOR (95% CI)		Made a suicide plan AOR (95% CI)		Attempted suicide AOR (95% CI)		Injurious suicide attempt AOR (95% CI)	
	Model 1	Model 2	Model 1	Model 2	Model 1	Model 2	Model 1	Model 2
**Race/ethnicity** (ref = NL White)								
NL Black	**0.8**	0.9	1.0	**1.2**	**1.6**	**1.8**	**1.7**	**2.0**
	**(0.7, 0.9)**	(0.8, 1.1)	(0.8, 1.2)	**(1.1, 1.9)**	**(1.3, 2.0)**	**(1.4, 2.3)**	**(1.2, 2.5)**	**(1.4, 3.0)**
Latinx	**0.8**	**0.7**	0.8	0.8	1.0	1.1	1.4	1.5
	**(0.7, 0.9)**	**(0.6, 0.8)**	(0.7, 1.0)	(0.6, 1.0)	(0.8, 1.4)	(0.7, 1.5)	(0.9, 2.2)	(0.8, 2.7)
NL Asian	1.0	**1.3**	1.1	**1.5**	0.9	1.3	1.1	1.7
	(0.8, 1.2)	**(1.0, 1.6)**	(0.9, 1.4)	**(1.2, 1.9)**	(0.6, 1.4)	(0.9, 2.1)	(0.6, 2.1)	(0.9, 3.3)
Multi- Latinx	1.0	0.9	1.1	1.0	**1.3**	**1.2**	**1.5**	**1.4**
	(0.9, 1.2)	(0.8, 1.0)	(0.9, 1.3)	(0.8, 1.2)	**(1.1, 1.6)**	**(1.0, 1.5)**	**(1.0, 2.1)**	**(1.0, 2.0)**
Multi- NL	**1.4**	**1.2**	**1.6**	**1.4**	**1.8**	**1.6**	**1.9**	**1.8**
	**(1.2, 1.7)**	**(1.0, 1.5)**	**(1.3, 2.0)**	**(1.2, 1.7)**	**(1.4, 2.2)**	**(1.3, 2.0)**	**(1.1, 3.2)**	**(1.0, 3.2)**
**Gender** (ref = male)								
Female	**1.9**	**1.2**	**1.8**	**1.1**	**1.7**	1.1	**1.9**	1.2
	**(1.7, 2.2)**	**(1.1, 1.4)**	**(1.6, 2.0)**	**(1.0, 1.3)**	**(1.4, 2.0)**	(0.9, 1.3)	**(1.4, 2.6)**	(0.9, 1.7)
**Sexual identity** (ref = heterosexual)								
Lesbian, Gay, bisexual, or questioning	**3.7**	**2.6**	**3.4**	**2.4**	**3.4**	**2.2**	**2.9**	**1.7**
	**(3.2, 4.3)**	**(2.3, 3.1)**	**(3.0, 4.0)**	**(2.1, 2.8)**	**(2.8, 4.2)**	**(1.8, 2.7)**	**(2.0, 4.1)**	**(1.2, 2.5)**
**Receipt of good grades (A’s or B’s)**		**0.8**		**0.7**		**0.6**		**0.5**
		**(0.7, 0.9)**		**(0.6, 0.8)**		**(0.5, 0.7)**		**(0.4, 0.7)**
**Bullied on school property**		**1.8**		**1.8**		**1.8**		**1.7**
						**(1.5, 2.2)**		**(1.3, 2.3)**
		**(1.5, 2.1)**		**(1.5, 2.1)**				
**Electronically bullied**		**1.5**		**1.5**		**1.8**		**2.3**
				**(1.2, 1.7)**		**(1.5, 2.2)**		**(1.7, 3.1)**
		**(1.3, 1.8)**						
**Feeling sad**		**11.4**		**9.0**		**8.8**		**8.7**
		**(9.8, 13.2)**		**(7.8, 10.3)**		**(7.0, 11.1)**		**(5.6, 13.4)**
**Having a good night sleep**		**0.7**		**0.7**		**0.7**		0.8
		**(0.6, 0.8)**		**(0.6, 0.8)**		**(0.6, 0.9)**		(0.6, 1.2)
**Year 2019 (vs. 2017)**	**0.9**	**0.8**	0.9	0.9	0.9	0.9	0.8	0.9
	**(0.8, 1.0)**	**(0.7, 0.9)**	(0.8, 1.1)	(0.8, 1.0)	(0.8, 1.2)	(0.8, 1.1)	(0.6, 1.1)	(0.6, 1.2)
**Total N**	**26639**	**22891**	**26631**	**22885**	**20273**	**19166**	**18584**	**17629**

*adjusted for grade level

## Discussion

Our results highlight race/ethnicity disparities in suicide ideation/behavior among understudied minorities including multi-racial and Asian groups. We found that among all groups multi-racial non-Latinx students experienced the highest rates of suicide ideations and behaviors, suggesting the influence of unique cultural influences and discrimination experienced by this group [23]. Asian American students were more likely to have suicidal thoughts and make plans compared to non-Latinx White students suggesting that Asian students may be at higher risk of attempting suicides, although this requires further investigation.

Multiracial groups consistently exhibited higher odds of suicidal ideation and behaviors. This trend could be attributed to disparities experienced by multiracial adolescents in substance use, particularly with illicit drugs such as marijuana and heroin, as well as in depressed mood [24].

Our findings, indicating that Asian high school students have similar odds of suicidal behaviors as White students, parallel a study examining racial and ethnic minority middle school students [25]. Our study extends this understanding to high school students, providing a more comprehensive picture of the issue across different age groups. Our study adds that while the prevalence of suicidal behaviors is similar between Asian and White students, Asian students do have higher odds of experiencing suicidal ideation and making suicide plans compared to White students.

These results also suggest the need to examine ways to better inform prevention and intervention efforts among understudied racial subgroups and to ensure equitable reporting or suicidal risk among understudied populations. Western psychology and methods have long dominated suicide counseling techniques across schools and communities in the U.S. The high rates of suicide ideations and behaviors among non-Latinx students as well as the elevated risk of suicidal thoughts and plans among Asian American students suggests that the predominant western psychiatric model may not be the best fit for the crisis of suicide for many students [26]. Schools and communities may benefit from cultural adaptations of evidence-based prevention programs that draw from culturally grounded methods in the design and development or prevention programs for specific populations and communities [27]. Future studies should examine the needs facing these emerging and growing populations as well as study subgroup differences within minority groups using a full continuum community based participatory or qualitative research methods to inform the development of interventions from a “ground up” or adaptive approach [28, 29].

This study has several limitations. There is a potential for underreported data due to self-reporting bias. YRBS only surveyed high school students, suggesting that the study sample is not representative of all youth in this age group. Furthermore, due to limited sample size, the study did not address all minority subgroups available in the YRBS and disaggregated race into ethnicity. As a result, our findings may mask critical sub-group differences and disparities. Additionally, we recognize that the post-COVID era may have introduced unique stressors and challenges for adolescents, potentially impacting their mental health and suicide behavior. Finally, there may be other potential confounding variables not included here, such as other mental health illnesses or immigration status.

## Conclusion

Suicide is increasing in the United States among high school students with widening racial/ethnic disparities. Prior studies examining this topic excluded fast-growing minority groups such as multi-racial youth–the same group that experienced the highest risk of suicide ideation and behaviors. More research is needed to understand the needs of understudied youth groups and inform targeted intervention efforts.

## Supporting information

S1 Table Study sample characteristics. (DOCX)
