# Supplementary material for: Suicide ideation and behavior disparities among high school students: Examining Asian and multiracial race/ethnicity groups
Source: PLOS Ment Health. 2024 Jul 2;1(2):e0000052. doi: 10.1371/journal.pmen.0000052 (PMC12798501; doi:10.1371/journal.pmen.0000052)
Supplement: S1 Table — (DOCX) [file pmen.0000052.s001.docx]

**S1. Study sample characteristics***

| Sociodemographic/racial-ethnic variables | Weighted %  (SE) | N |
| --- | --- | --- |
| Female | 50.0  (0.008) | 12873 |
| 9^th^ grade | 27.0  (0.005) | 7042 |
| 10^th^ grade | 25.6  (0.003) | 7052 |
| 11^th^ grade | 24.2  (0.003) | 6571 |
| 12^th^ grade | 23.5  (0.004) | 5974 |
| Lesbian, Gay, bisexual or questioning | 7.0  (0.009) | 1909 |
| NL Asian | 4.3  (0.007) | 1242 |
| NL Black | 13.0  (0.009) | 4727 |
| NL White | 53.0  (0.018) | 12705 |
| Latinx | 9.6  (0.008) | 2511 |
| Multiracial-Latinx | 15.0  (0.011) | 4001 |
| Multiracial-NL | 5.1  (0.003) | 1453 |
| Suicide ideation | 18.0  (0.004) | 4876 |
| Made a suicide plan** (n=26631) | 14.4  (0.004) | 3903 |
| Suicide attempt** (n=20273) | 7.9  (0.004) | 1747 |
| Injurious suicide attempt** (18584) | 2.4  (0.002) | 466 |
| n |  | 26639 |

*Sample population estimates based on respondents who answered suicide ideation question without missing race/ethnicity data

**Different sample size-based students who have indicated they participate in a selected activity or behavior
